# Generalized pustular psoriasis successfully treated with spesolimab in the setting of metastatic colon cancer

**DOI:** 10.1016/j.jdcr.2024.05.016

**Published:** 2024-05-19

**Authors:** Diamond Rose Guy, Sydney DeVore, Vatsala Kirtani, Nananamibia Duffy

**Affiliations:** a University of Rochester School of Medicine and Dentistry, Rochester, New York; b University of Pittsburgh School of Medicine, Pittsburgh, Pennsylvania; c Division of Hematology and Oncology, Rochester Regional Health, Rochester, New York; d Division of Dermatology, Rochester Regional Health, Rochester, New York

**Keywords:** cancer, generalized pustular psoriasis, metastatic, spesolimab

## Introduction

Generalized pustular psoriasis (GPP) is a rare, life-threatening subtype of pustular psoriasis characterized by recurrent eruptions of widespread epidermal neutrophilic sterile pustules.^1^ The pathogenesis of GPP has not yet been fully elucidated; nonetheless, there is an interplay between environmental and genetic factors. Environmental risk factors include antimicrobial drugs, infection, pregnancy, stress, withdrawal from corticosteroids, and prior psoriasis vulgaris.^2^ Genetically, mutations in the interleukin-36 (IL-36) signaling pathway have a unique and major role in GPP, as IL-36 dysregulation commences excess production of cytokines and autoinflammation. Studies have shown that these IL-36 mutations occur more in people of Asian descent.^1^ Spesolimab is a humanized monoclonal antibody that binds directly to the IL-36 receptor and blocks downstream signaling.^3^ In September 2022, spesolimab was approved by the United States Food and Drug Administration as the first treatment option for GPP.^4^ Here, we present a unique case of GPP that significantly improved with spesolimab in the setting of metastatic colon cancer.

## Case report

The patient is a 76-year-old Asian gentleman and practicing physician with a medical history significant for chronic renal disease, chronic plaque psoriasis, and metastatic adenocarcinoma of the sigmoid colon. He presented to our clinic with a diffuse rash over his trunk. Prior to this eruption, he received two infusions of FLOFOX (folinic acid + 5-fluorouracil + oxaliplatin) for his metastatic colon cancer, as he was not a surgical candidate. The patient also received 12 mg of dexamethasone with each infusion.

**Fig 1:**
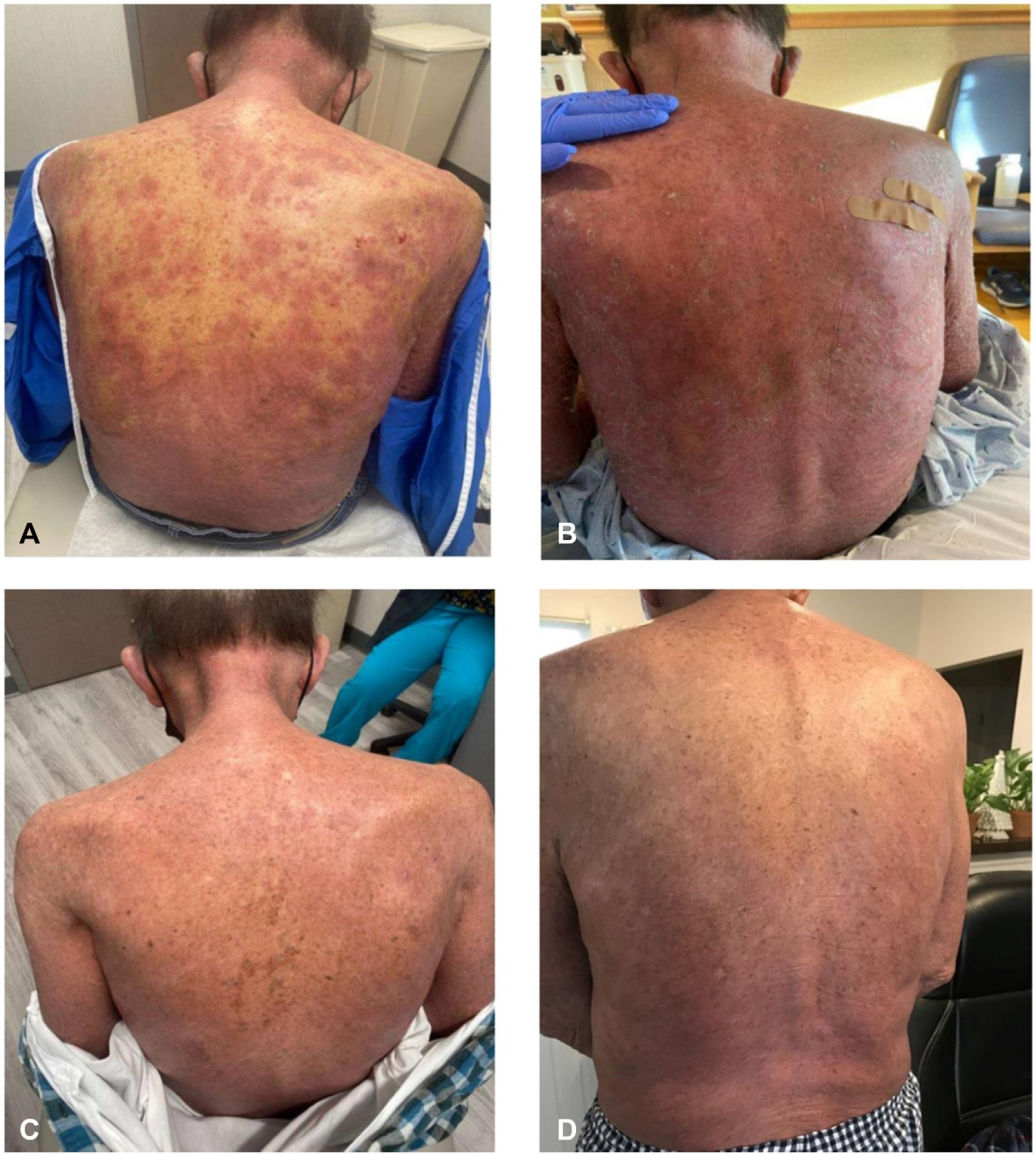
Initial presentation, progression, and resolution of GPP in a patient with metastatic colon cancer treated with one infusion of spesolimab. **A,** Initial presentation of his breakthrough GPP. **B,** Progression to widespread erythroderma just prior to spesolimab infusion. **C,** Approximately ∼90% clearance of his GPP 4 days post-spesolimab infusion. **D,** Approximately 100% clearance of his GPP 7 days post-spesolimab infusion. *GPP*, Generalized pustular psoriasis.

In the exam room, the patient was tremulous and tachycardic; a space heater and blankets were brought in for his comfort. On a physical exam, there were widespread, pink, round plaques studded with pustules, many coalescing into broad areas of erythema, scaling, and pustules (Fig 1, *A*). Based on his skin exam and history, the diagnosis of pustular psoriasis was suspected. Two punch biopsies from the right upper back were performed. However, immediate treatment was required. We decided against cyclosporine given our patient’s baseline creatinine level of 3 mg/dL. The decision to start spesolimab was made. Careful consideration was given to the fact that our patient was undergoing chemotherapy for an active malignancy, but it was determined that the benefits of spesolimab outweighed the risks. We were unsure how soon this drug would arrive, as this was the first time it had been prescribed in our region. Therefore, we decided to inject secukinumab 300 mg subcutaneously. The patient was also started on cefadroxil 500 mg twice daily empirically after obtaining bacterial cultures. QuantiFERON gold was ordered. Viral cultures were also obtained, and the patient was empirically started on valacyclovir.

**Fig 2:**
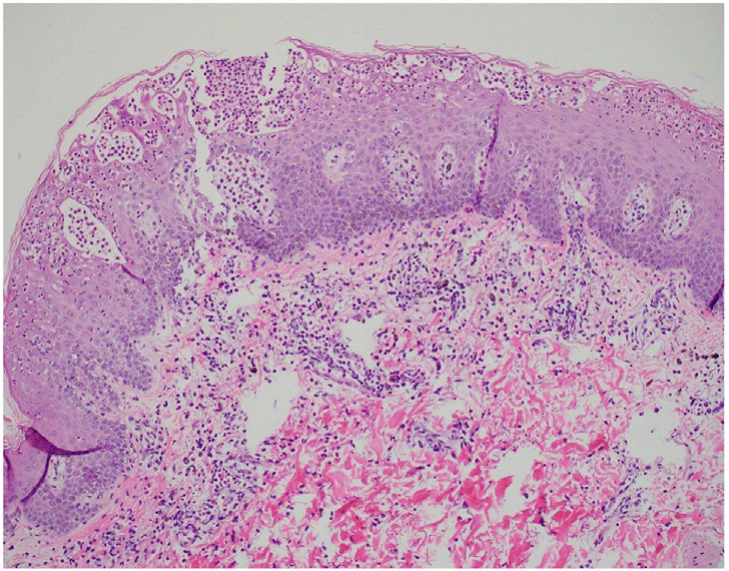
Pathology specimen exhibited mild hyperkeratosis, focal scale crust with neutrophils, irregular epidermal hyperplasia, multifocal intracorneal, and intraepidermal spongiform. Pustules contain neutrophils, acantholytic keratinocytes, and cell debris. Spongiosis with extensive neutrophilic exocytosis, focal early acantholysis, edema of the papillary and superficial reticular dermis, dilated vessels, focal extravasated erythrocytes, and numerous superficial and deep perivascular and interstitial pigment-laden macrophages. Moderately dense superficial and mid-dermal perivascular with interstitial mixed inflammatory infiltrates of neutrophils, histiocytes, lymphocytes, and occasional eosinophils and plasma cells.

While this drug can be administered in an outpatient infusion center, because of the patient's underlying malignancy, we felt it was prudent for him to be admitted to the hospital for his infusions. He was admitted to an oncology floor in preparation for his spesolimab infusion. Prior to the infusion, it was noted that the patient’s rash had spread and he was nearly erythrodermic (Fig 1, *B*). A single intravenous infusion of spesolimab was administered over 90 minutes. By this time, the results from the skin biopsies and cultures had been released. Biopsies were consistent with pustular dermatitis, and the diagnosis of GPP was made (Fig 2). Bacterial cultures only grew mixed flora. These results confirmed our suspicion of GPP.

Two days later, our patient was discharged home with triamcinolone 0.1% ointment. Notably, he tolerated spesolimab well without any adverse events. During that admission, the patient’s vital signs, complete blood count, and comprehensive metabolic panel were monitored daily for any signs of adverse events.

Four days post-spesolimab, his pustular psoriasis was ∼90% clear (Fig 1, *C*). Photographs in the subsequent days showed nearly 100% clearance of his psoriasis (Fig 1, *D*). Eventually, he restarted acitretin 25 mg once daily, and has been cleared of his psoriasis ever since; except for a couple of small scaly plaques on his abdomen. In an effort to reduce the risk of systemic steroids coadministered with chemotherapy, his oncologist changed his regimen from FLOFOX to FOLFIRI (folinic acid + 5-fluorouracil + irinotecan) 21 days post-spesolimab infusion. FOLFIRI was administered every 14 days for 10 cycles (cycle 3 was delayed due to myelosuppression).

Two months post-spesolimab, follow-up positron emission tomography scan revealed complete resolution of all cancerous activity (a previously noted primary lesion in his sigmoid colon and throughout his liver). Three months later, post-spesolimab, the patient had a few scaly plaques on his trunk but was continued on acitretin 25 mg once daily. Six months post-spesolimab, his GPP flared on his trunk, and acitretin was increased to 50 mg once daily. A repeat positron emission tomography scan obtained that same week revealed progression of the sigmoid mass and recurrence of metastatic lesions in the liver. The patient’s oncologist decided to restart his FLOFOX and dexamethasone regimen. Seven months post-spesolimab, this patient’s GPP improved, and he continued acitretin 50 mg once daily. One year and 3 months after the patient's single infusion of spesolimab, he remains stable with his psoriasis.

## Discussion

The efficacy of spesolimab in treating GPP is well documented in the literature.^5^^,^^6^ The significant resolution of GPP in our patient with just one infusion supports the efficacy of this drug. This case is unique as spesolimab was started amid chemotherapy for active metastatic colon cancer. To our knowledge, treatment of GPP with spesolimab in the presence of active cancer metastases has not been previously reported. Patients with active malignancy or a history of malignancy in the past 5 years (except for treated nonmelanoma skin cancers or in-situ cancer of the cervix) were excluded from the clinical trials that brought spesolimab to market.^7^ This case report highlights both the safety and efficacy of spesolimab in a patient with active malignancy.

## Conflicts of interest

Dr Duffy has served on an advisory board with Janssen and has been a consultant for Boehringer Ingelheim (2 years after the patient was treated). Drs Kirtani, DeVore, and Guy have no conflicts of interest to declare.
